# Sargramostim to treat patients with acute hypoxic respiratory failure due to COVID-19 (SARPAC): A structured summary of a study protocol for a randomised controlled trial

**DOI:** 10.1186/s13063-020-04451-7

**Published:** 2020-06-05

**Authors:** Cedric Bosteels, Bastiaan Maes, Karel Van Damme, Elisabeth De Leeuw, Jozefien Declercq, Anja Delporte, Bénédicte Demeyere, Stéfanie Vermeersch, Marnik Vuylsteke, Joren Willaert, Laura Bollé, Yuri Vanbiervliet, Jana Decuypere, Frederick Libeer, Stefaan Vandecasteele, Isabelle Peene, Bart Lambrecht

**Affiliations:** VIB-UGent Inflammatie-researchcentrum, Oost-Vlaanderen, Ghent, Belgium

**Keywords:** COVID-19, Randomised controlled trial, protocol, sargramostim, GM-CSF, leukine®, oxygenation, hypoxic failure, PF ratio, A-a gradient, inflammatory monocyte, alveolar macrophage

## Abstract

**Objectives:**

The hypothesis of the proposed intervention is that Granulocyte-macrophage colony-stimulating factor (GM-CSF) has profound effects on antiviral immunity, and can provide the stimulus to restore immune homeostasis in the lung with acute lung injury post COVID-19, and can promote lung repair mechanisms, that lead to a 25% improvement in lung oxygenation parameters. Sargramostim is a man-made form of the naturally-occurring protein GM-CSF.

**Trial design:**

A phase 4 academic, prospective, 2 arm (1:1 ratio), randomized, open-label, controlled trial.

**Participants:**

Patients aged 18-80 years admitted to specialized COVID-19 wards in 5 Belgian hospitals with recent (< 2 weeks prior to randomization) confirmed COVID-19 infection and acute respiratory failure defined as a PaO2/FiO2 below 350 mmHg or SpO2 below 93% on minimal 2 L/min supplemental oxygen. Patients were excluded from the trial in case of (1) known serious allergic reactions to yeast-derived products, (2) lithium carbonate therapy, (3) mechanical ventilation prior to randomization, (4) peripheral white blood cell count above 25.000/μL and/or active myeloid malignancy, (5) high dose systemic steroid therapy (> 20 mg methylprednisolone or equivalent), (6) enrolment in another investigational study, (7) pregnant or breastfeeding or (8) ferritin levels > 2000 μg/mL.

**Intervention and comparator:**

Inhaled sargramostim 125 μg twice daily for 5 days in addition to standard care. Upon progression of disease requiring mechanical ventilation or to acute respiratory distress syndrome (ARDS) and initiation of mechanical ventilator support within the 5 day period, inhaled sargramostim will be replaced by intravenous sargramostim 125 μg/m^2^ body surface area once daily until the 5 day period is reached. From day 6 onwards, progressive patients in the active group will have the option to receive an additional 5 days of IV sargramostim, based on the treating physician's assessment. Intervention will be compared to standard of care. Subjects progressing to ARDS and requiring invasive mechanical ventilatory support, from day 6 onwards in the standard of care group will have the option (clinician's decision) to initiate IV sargramostim 125m μg/m^2^ body surface area once daily for 5 days.

**Main outcomes:**

The primary endpoint of this intervention is measuring oxygenation after 5 days of inhaled (and intravenous) treatment through assessment of a change in pretreatment and post-treatment ratio of PaO2/FiO2 and through measurement of the P(A-a)O2 gradient (PAO2= Partial alveolar pressure of oxygen, PaO2=Partial arterial pressure of oxygen; FiO2= Fraction of inspired oxygen).

**Randomisation:**

Patients will be randomized in a 1:1 ratio. Randomization will be done using REDCap (electronic IWRS system).

**Blinding (masking):**

In this open-label trial neither participants, caregivers, nor those assessing the outcomes will be blinded to group assignment.

**Numbers to be randomised (sample size):**

A total of 80 patients with confirmed COVID-19 and acute hypoxic respiratory failure will be enrolled, 40 in the active and 40 in the control group.

**Trial Status:**

SARPAC protocol Version 2.0 (April 15 2020). Participant recruitment is ongoing in 5 Belgian Hospitals (i.e. University Hospital Ghent, AZ Sint-Jan Bruges, AZ Delta Roeselare, University Hospital Brussels and ZNA Middelheim Antwerp). Participant recruitment started on March 26^th^ 2020. Given the current decline of the COVID-19 pandemic in Belgium, it is difficult to anticipate the rate of participant recruitment.

**Trial registration:**

The trial was registered on Clinical Trials.gov on March 30^th^, 2020 (ClinicalTrials.gov Identifier: NCT04326920) - retrospectively registered; https://clinicaltrials.gov/ct2/show/NCT04326920?term=sarpac&recrs=ab&draw=2&rank=1 and on EudraCT on March 24th, 2020 (Identifier: 2020-001254-22).

**Full protocol:**

The full protocol is attached as an additional file, accessible from the Trials website (Additional file 1). In the interest in expediting dissemination of this material, the familiar formatting has been eliminated; this Letter serves as a summary of the key elements of the full protocol.

## Supplementary information

**Additional file 1.** Full study protocol.

## Data Availability

Not applicable.

