# Correction to: Sargramostim to treat patients with acute hypoxic respiratory failure due to COVID-19 (SARPAC): A structured summary of a study protocol for a randomised controlled trial

**DOI:** 10.1186/s13063-020-04520-x

**Published:** 2020-06-22

**Authors:** Cedric Bosteels, Bastiaan Maes, Karel Van Damme, Elisabeth De Leeuw, Jozefien Declercq, Anja Delporte, Bénédicte Demeyere, Stéfanie Vermeersch, Marnik Vuylsteke, Joren Willaert, Laura Bollé, Yuri Vanbiervliet, Jana Decuypere, Frederick Libeer, Stefaan Vandecasteele, Isabelle Peene, Bart N. Lambrecht

**Affiliations:** VIB-UGent Inflammatie-researchcentrum, Oost-Vlaanderen, Ghent, Belgium

**Correction to: Trials (2020) 21:49**

**https://doi.org/10.1186/s13063-020-04451-7**

Following publication of the original article [1], we have been notified that an initial was missed from one of the author names.

Originally published author names:

- Bart Lambrecht

Correct author names:

- Bart N. Lambrecht

The original article has been corrected.
