# Supplementary material for: Sargramostim to treat patients with acute hypoxic respiratory failure due to COVID-19 (SARPAC): A structured summary of a study protocol for a randomised controlled trial
Source: Trials. 2020 Jun 5;21:491. doi: 10.1186/s13063-020-04451-7 (PMC7273817; doi:10.1186/s13063-020-04451-7)
Supplement: Supplementary file 1 — Additional file 1. Full study protocol. [file 13063_2020_4451_MOESM1_ESM.pdf]

# A prospective, randomized, open-label, interventional study to investigate the efficacy of sargramostim (Leukine®) in improving oxygenation and short- and long-term outcome of COVID-19 patients with acute hypoxic respiratory failure.

Sargramostim in patients with acute hypoxic respiratory failure due to COVID-19 (SARPAC)

| | |
|---|---|
| Acronym / Protocol code | SARPAC |
| Protocol version and date | 2.0 15APR2020 |
| Phase | 4 |
| EudraCT n° | 2020-001254-22 |
| Sponsor | University Hospital Ghent,<br>C. Heymanslaan 10<br>9000 Ghent<br>Belgium |
| Financial/Material Support: | Partner Therapeutics will provide sargramostim |

Coordinating Investigator:

**Bart N. Lambrecht, MD, PhD**
**Department of Internal Medicine & Pediatrics,**
**Department of Respiratory Medicine**
**University Hospital Ghent**
**Corneel Heymanslaan**
**9000 Ghent, Belgium**
**+32/93329110**

Co-investigators: Multicenter Trial in Belgium

A prospective, randomized, open-label, interventional study to investigate the efficacy of sargramostim (Leukine®) in improving oxygenation and short- and long-term outcome of COVID-19 patients with acute hypoxic respiratory failure.

Sargramostim in patients with acute hypoxic respiratory failure due to COVID-19 (SARPAC)

# Protocol Co-ordinating Investigator signature page

I certify that I will conduct the study in compliance with the protocol, any amendments, GCP and the declaration of Helsinki, and all applicable regulatory requirements.

**Investigator:**

Name: Prof. Dr. B. Lambrecht
Function: Pulmonologist
Institution: UZ Ghent

**Date:** **Signature:**

A prospective, randomized, open-label, interventional study to investigate the efficacy of sargramostim (Leukine®) in improving oxygenation and short- and long-term outcome of COVID-19 patients with acute hypoxic respiratory failure.

Sargramostim in patients with acute hypoxic respiratory failure due to COVID-19 (SARPAC)

# Protocol Site Principal Investigator signature page

I certify that I will conduct the study in compliance with the protocol, any amendments, GCP and the declaration of Helsinki, and all applicable regulatory requirements.

**Investigator:**

Name: Prof. Dr. B. Lambrecht
Function: Pulmonologist
Institution: UZ Ghent

**Date:** **Signature:**

## Protocol Amendment History:

| Version Number | Date | Description of amendment |
|---|---|---|
| **1.4** | **10APR2020** | Specification of participating centers (UZ Ghent and AZ Sint Jan Brugge to multicenter trial |
| | | P31-34: extra sampling only in selected centers |
| 2.0 | 15APR2020 | p. 12, 26: Inclusion criteria 1 removed and changed to COVID-19 diagnosis confirmed by antigen detection test and/or PCR and/or positive serology, or any emerging and validated diagnostic laboratory test for COVID-19 within this period. |
| | | p. 12, 26 Extra Inclusion criteria: In some patients, it may be impossible to get a confident laboratory confirmation of COVID-19 diagnosis after 24h of hospital admission because viral load is low and/or problems with diagnostic sensitivity. In those cases, in absence of an alternative diagnosis, and with highly suspect bilateral ground glass opacities on recent (<24h) chest-CT scan (confirmed by a radiologist and pulmonary physician as probable COVID-19), a patient can be enrolled as probable COVID-19 infected. In all cases, this needs confirmation by later seroconversion |
| | | Page 36: redefining sampling (10.) due to addition of extra study sites.<br>Page 31, 33, 34, 35 : better definition of duration of treatment<br>Page 42 : Despite the known safety profile of the study medications and study design, a DSMB is foreseen. |
| | | Page 15, 21, 22, 25, 26, 28, 31, 33. Better definition of progressive disease: Progression to ARDS requiring mechanical ventilation is removed and replaced by: progressive disease requiring mechanical ventilatory support. |
| | | Page 22, 24, 34. Safety follow-up period is 10-20 weeks. |
| | | Page 13,30 : Nebulizing is preferably done in an isolation negative pressure chamber, and if not, personnel should use an FFP2 mask. Patient should self-administer the medication and where possible, the room should not be entered within one hour after administration. |
| | | Page 35: arterial blood gas mandatory at D1, D6 and FU<br>Page 33; 35: if arterial blood gas is taken within 24h before first dose administration, as described in point° the arterial blood gas of screening can be used as D1 value |
| | | Page 29, 35: If a patient decides to leave hospital before day 6 of the study, for example because of clinical improvement, the oxygenation parameters at day of discharge will be used to calculate the primary endpoint measurement. |

# TABLE OF CONTENTS

LIST OF ABBREVIATIONS ..... 9
1. Protocol Summary ..... 10
1.1. Protocol specifics ..... 11
1.2. Study Type and Study Phase ..... 11
1.3. Aim of the study (including primary endpoints) ..... 11
1.4. Subjects ..... 11
1.4.1. Number of subjects ..... 11
1.4.2. Target group ..... 11
1.5. Inclusion and exclusion criteria ..... 11
1.6. Study Interventions ..... 12
1.6.1. IMPs and dosage ..... 13
1.6.2. Schematic overview of the data collection & interventions ..... 14
1.7. Study duration ..... 14
2. Rationale and background ..... 15
2.1. Rationale ..... 15
2.2. Background ..... 17
2.3. Risk/Benefit Assessment ..... 19
2.4. Limitations ..... 19
3. Objectives ..... 20
3.1. Primary Objectives ..... 20
3.2. Secondary Objectives ..... 21
4. End Points + Time Points ..... 21
4.1. Primary End Points + Time Points ..... 21
4.2. Secondary End Points + Time Points ..... 22
5. Study design ..... 24
5.1. Description of study design ..... 24
5.2. End of Study Definition ..... 25
5.2.1. For an individual subject ..... 25
5.2.2. For the whole study ..... 25
5.3. Estimated duration of the study ..... 25
6. Inclusion and Exclusion Criteria ..... 26
6.1. Inclusion Criteria ..... 26

6.2. Exclusion Criteria ........ 26
6.2.1. Screen failures ........ 27
7. Target Population ........ 28
7.1. Subjects ........ 28
7.1.1. Number of subjects and planned recruitment rate ........ 28
7.1.2. Withdrawal and replacement of subjects ........ 28
7.2. Method of recruitment ........ 29
7.3. Screening ........ 29
8. Investigational Medicinal Product (IMP) ........ 30
8.1. Name of the IMP ........ 30
8.1.1. Composition and active substance of the IMP ........ 30
8.1.2. Producer and Distributor of the IMP ........ 30
8.1.3. Preparation + Dosage + administration of the IMP ........ 30
8.1.4. Permitted dose adjustments and interruption of treatment ........ 31
8.1.5. Duration of treatment ........ 31
8.1.6. Packaging and Labeling of the IMP ........ 31
8.1.7. Storage conditions of the IMP ........ 31
8.1.8. Known side effects of the medication ........ 31
8.2. Concomitant / Rescue Medication ........ 32
9. Study Specific Procedures ........ 33
9.1. Randomisation ........ 33
9.2. Study specific interventions ........ 33
9.3. Overview of collected data ........ 34
9.4. Schematic overview of the data collection & interventions ........ 35
9.5. Restrictions for subjects during the study ........ 35
10. Sampling ........ 36
10.1. Types and number of samples ........ 36
10.2. Timepoints of sampling ........ 36
10.3. Sample Handling & Analysis ........ 36
10.4. Sample Storage and/or shipping ........ 36
10.5. Future use of stored samples ........ 36
11. Statistical Considerations ........ 37
11.1. Sample size calculation ........ 37
11.2. Type of statistical methods ........ 37
11.3. Statistical analysis team ........ 37

12. Data handling ... 37
12.1. Method of data collection ... 37
12.1.1. Case Report Form ... 37
12.1.2. Data directly collected in the CRF (no source available) ... 38
12.2. Data storage ... 38
12.3. Archiving of data ... 38
12.4. Access to data ... 38
13. Safety ... 39
13.1. Definitions ... 39
13.2. Reporting requirements ... 40
13.2.1. AE reporting ... 40
13.2.2. SAE reporting ... 40
13.2.3. SUSAR reporting ... 41
13.3. List of contact details for safety reporting ... 41
13.4. Flowchart Reporting ... 41
13.5. Events, excluded from reporting ... 42
13.6. Data Safety Monitoring Board (DSMB) ... 42
13.7. Development Safety Update Report ... 42
14. Monitoring/Auditing/Inspection ... 43
14.1. Monitoring ... 43
14.1.1. General ... 43
14.1.2. Monitoring team ... 43
14.1.3. Scope ... 43
14.2. Inspection ... 43
14.3. Protocol Deviation policy ... 43
14.4. Serious breach to GCP and/or the protocol ... 43
15. Ethical and legal aspects ... 44
15.1. Good Clinical Practice ... 44
15.2. Informed Consent ... 44
15.3. Approval of the study protocol ... 44
15.3.1. General ... 44
15.3.2. Protocol amendments ... 45
15.4. Confidentiality and Data Protection ... 45
15.5. Liability and Insurance ... 45
15.6. End of Study Notification ... 46

16. Publication policy ........................................ 47
17. Reference List ........................................ 47
Appendices ........................................ 49
Appendix 1: USPI (US Package insert)........................................ 49

# LIST OF ABBREVIATIONS

| | | |
|---|---|---|
| AE | = | Adverse Event |
| AECC | = | American-European Consensus Conference |
| ARDS | = | Acute Respiratory Distress Syndrome |
| CI | = | Coordinating Investigator |
| COVID-19 | = | Coronavirus induced disease-2019 |
| CT | = | Clinical Trial Unit |
| DSMB | = | Data Safety Monitoring Board |
| DSUR | = | Development Safety Update Report |
| EC | = | Ethics Committee |
| ECG | = | Electrocardiogram |
| eCRF | = | electronic Case Report Form |
| EDC | = | Electronic Data Capture |
| EPD | = | Electronic Patient Dossier |
| FAMHP | = | Federal Agency for Medicines and Health Products |
| FiO2 | = | Fraction of inspired oxygen |
| FPI | = | First Patient In |
| FVC | = | Forced vital capacity |
| GCP | = | Good Clinical Practice |
| GDPR | = | General Data Protection Regulation |
| GM-CSF | = | Granulocyte-macrophage colony stimulating factor |
| GMP | = | Good Manufacturing Practice |
| HIRUZ | = | Health, Innovation and Research Institute UZ Ghent |
| HLH | = | Hyperferritinemia and Hemophagocytic Lymphohistiocytosis |
| IB | = | Investigator's Brochure |
| ICF | = | Informed Consent Form |
| ICH | = | International Council for Harmonisation |
| IMP | = | Investigational Medicinal Product |
| IMPD | = | Investigational Medicinal Product Dossier |
| LVLS | = | Last Visit, Last Subject |
| PCWP | = | Pulmonary Capillary Wedge Pressure |
| PEEP | = | Positive End Expiratory Pressure |
| PI | = | Principal Investigator |
| $PaO_2$ | = | Partial pressure of oxygen |
| SAE | = | Serious Adverse Event |
| sHLH | = | secondary hemophagocytic lymphohistiocytosis |
| SmPC | = | Summary of Product Characteristics |
| SOP | = | Standard Operating Procedure |
| SUSAR | = | Suspected Unexpected Serious Adverse Reaction |
| TERENA | = | Trans-European Research and Education Networking Association |
| TLC | = | Total Lung Capacity |
| TLS | = | Transport Layer Security |

## 1. Protocol Summary

**SARPAC trial** : Use of sargramostim in patients with acute hypoxic respiratory failure due to COVID-19

| | |
|---|---|
| Title | A prospective, randomized, open-label, interventional study to investigate the efficacy of sargramostim (Leukine®) in improving oxygenation and short- and long-term outcome of COVID-19 patients with acute hypoxic respiratory failure. |
| Protocol number | SARPAC |
| Protocol version | V2.0 |
| EudraCT number | 2020-001254-22 |
| Sponsor | University Hospital Ghent |
| Co-ordinating Investigator | Bart N. Lambrecht |
| Type of study | Interventional |
| Fase | IV |
| Methodology | prospective, randomized, open-label study |
| Study duration | 22 weeks |
| Purpose of study | To study the effectiveness of additional sargramostim (GM-CSF) inhalation versus standard of care on blood oxygenation in patients with COVID-19 coronavirus infection and acute hypoxic respiratory failure |
| Number of participants | 80 |
| Study population and main inclusion criteria | Patients with confirmed COVID-19 infection and acute hypoxic respiratory failure<br>Presence of hypoxic respiratory failure defined as $O_2$ saturation below 93% on minimal 2l/min $O_2$ therapy and/or ratio $PaO_2/FiO_2$ below 350 |
| Investigational drug, dose, route | Sargramostim/Leukine® 125 mcg BID via inhalation, for 5 days<br>Sargramostim/Leukine® 125 mcg/m2 once daily IV upon progression, for 5 days |
| Treatment duration | 5 days, followed by possible 5 day extension upon deterioration |

## 1.1. Protocol specifics

EudraCT number : 2020-001254-22

**University Hospital Ghent**

## 1.2. Study Type and Study Phase

This is phase 4 academic, prospective, randomized, open-label, interventional study designed to investigate the efficacy of sargramostim (Leukine®) in improving oxygenation and short- and long-term outcome of COVID-19 patients with acute hypoxic respiratory failure.

## 1.3. Aim of the study (including primary endpoints)

The **primary objective** is to investigate whether the administration of inhaled sargramostim (Leukine®) at a dose of 250 mcg daily during 5 days improves oxygenation in COVID-19 patients with acute hypoxic respiratory failure.

The **secondary objectives** are:
- to study if early intervention with sargramostim is safe (number of AEs/SAEs)
- to study if early intervention with inhaled sargramostim affects clinical outcome defined by duration of hospital stay, 6-point ordinal scale, clinical sign score, SOFA score, NEWS2 score
- to study if early intervention with sargramostim affects the rate of nosocomial infection
- to study if early intervention with inhaled sargramostim affects progression to mechanical ventilation and/or ARDS
- to study if treatment with sargramostim affects all-cause mortality rate at 4 and 20 weeks post inclusion
-to study if treatment with sargramostim affects features of secondary haemophagocytic lymphohistiocytosis, defined by HS score
- to study if treatment with sargramostim has a favourable effect on long term 10-20 week follow up

## 1.4. Subjects

### 1.4.1. Number of subjects

A total of 80 patients with confirmed COVID-19 and acute hypoxic respiratory failure will be enrolled, 40 in the active and 40 in the control group.

### 1.4.2. Target group

Confirmed COVID-19 patients with acute hypoxic respiratory failure admitted to the COVID-19 isolation ward.

## 1.5. Inclusion and exclusion criteria

**Inclusion criteria**
The following patients will be enrolled:

- Recent (≤2weeks prior to randomization) confident diagnosis of COVID-19 confirmed by antigen detection and/or PCR, and/or seroconversion or any other emerging and validated diagnostic test.
- In some patients, it may be impossible to get a confident laboratory confirmation of COVID-19 diagnosis after 24h of hospital admission because viral load is low and/or problems with diagnostic sensitivity. In those cases, in absence of an alternative diagnosis, and with highly suspect bilateral ground glass opacities on recent (<24h) chest-CT scan (confirmed by a radiologist and pulmonary physician as probable COVID-19), a patient can be enrolled as probable COVID-19 infected. In all cases, this needs confirmation by later seroconversion.
- Presence of acute hypoxic respiratory failure defined as (either or both)
  - saturation below 93% on minimal 2 l/min O2
  - PaO2/FiO2 below 350

- Admitted to specialized COVID-19 ward
- Age 18-80
- Male or Female
- Willing to provide informed consent

**Exclusion criteria**
- Patients with known history of serious allergic reactions, including anaphylaxis, to human granulocyte-macrophage colony stimulating factor such as sargramostim, yeast-derived products, or any component of the product.
- mechanical ventilation before start of study
- patients with peripheral white blood cell count above 25.000 per microliter and/or active myeloid malignancy
-patients on high dose systemic steroids (> 20 mg methylprednisolone or equivalent)
-patients on lithium carbonate therapy
- Patients enrolled in another investigational drug study
- Pregnant or breastfeeding females (all female subjects regardless of childbearing potential status must have negative pregnancy test at screening)
- Patients with serum ferritin >2000 mcg/ml (which will exclude ongoing HLH)

## 1.6. Study Interventions

Confirmed or highly suspect COVID-19 patients with acute hypoxic respiratory failure (saturation below 93% on minimal 2 l/min O2 or PaO2/FiO2 <350) will be randomized to receive sargramostim 125mcg twice daily for 5 days as a nebulized inhalation on top of standard of care (active group), or to receive standard of care treatment (control group). Upon progression of disease requiring initiation of mechanical ventilatory support within the 5 day period, in patients in the active group, inhaled sargramostim will be replaced by intravenous sargramostim 125mcg/m$^2$ body surface area once daily until the 5 day period is reached. From day 6 onwards, progressive patients in the active group will have the option to receive an additional 5 days of IV sargramostim, based on the treating physician's assessment. In the control group with progressive disease requiring mechanical ventilatory support, from day 6 onwards, the treating physician will have the option to initiate IV sargramostim 125mcg/m$^2$ body surface area once daily for 5 days.
Safety data, including blood leukocyte counts, will be collected in all patients. Efficacy data will also be collected and will include arterial blood gases, oxygenation parameters, need for ventilation, lung compliance, organ function, radiographic changes, ferritin levels, triglyceride levels, etc. as well as occurrence of secondary bacterial infections.
Patients will stop the investigational drug if there is unacceptable toxicity according to investigator's judgement.

### 1.6.1. IMPs and dosage

**LEUKINE® (sargramostim) prepared and administered for inhalation using nebulizer**

LEUKINE for injection is a sterile, preservative-free lyophilized powder that requires reconstitution with 2mL normal saline solution. Once reconstituted, LEUKINE can be inhaled as an aqueous aerosol using either a vibrating mesh nebulizer (Philips InnospireGo) or jet nebulizer, per manufacturer instructions. (Nebulizers studied include: AKITA2 Apixneb, PARI LC-Plus set, PulmoAide, Pan LC, Aeroneb Solo Device). Use reconstituted LEUKINE® solution for inhalation within 16 hours following reconstitution and/or dilution.

Nebulizing is preferably done in an isolation negative pressure chamber, and if not, personnel should use an FFP2 mask. Patient should self-administer the medication and where possible, the room should not be entered within one hour after administration.

**LEUKINE® (sargramostim) prepared and administered intravenously**

For patients that are on a mechanical ventilator and cannot be treated with LEUKINE® inhalation:

- The recommended dose is 125 mcg/$m^2$/day administered intravenously over a 4-hour period once daily for up to 5 days.
- For intravenous injection: Administer LEUKINE injection in 0.9% Sodium Chloride Injection, USP. Dilute LEUKINE for intravenous infusion in 0.9% Sodium Chloride Injection, USP. If the final concentration of LEUKINE is below 10 mcg/mL, add Albumin (Human) at a final concentration of 0.1% to the saline prior to addition of LEUKINE to prevent adsorption to the components of the drug delivery system. To obtain a final concentration of 0.1% Albumin (Human), add 1 mg Albumin (Human) per 1 mL 0.9% Sodium Chloride Injection, USP (e.g., use 1 mL 5% Albumin [Human] in 50 mL 0.9% Sodium Chloride Injection, USP).

### 1.6.2. Schematic overview of the data collection & interventions

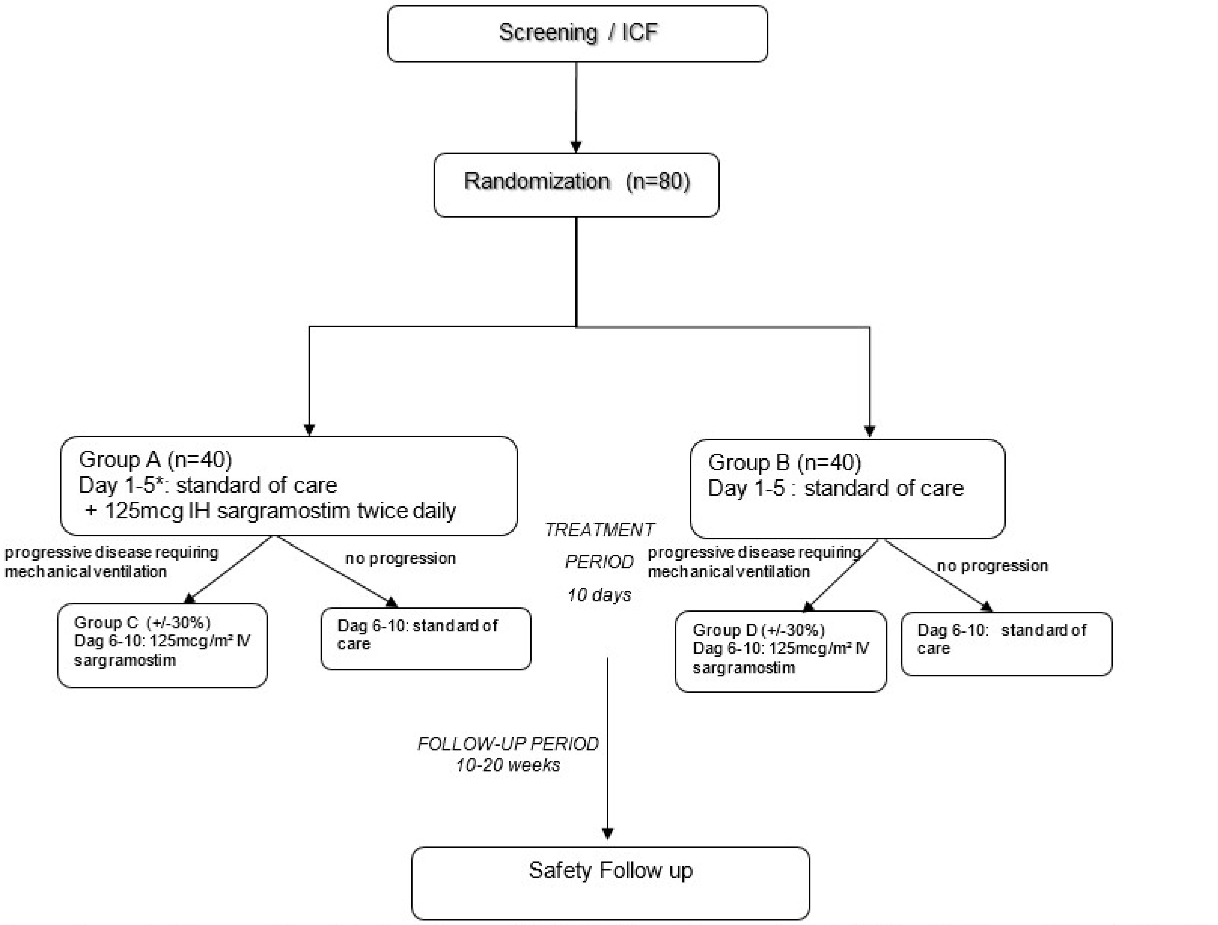


*in case of progressive disease requiring mechanical ventilator support within the first 5 days, IV sargramostim can be initiated until the 5 day period is reached. From day 6 onwards, progressive patients in the active group will have the option to receive an additional 5 days of IV sargramostim, based on the treating physician's assessment.

### 1.7. Study duration

The total treatment duration of the study is 10 days, and the entire study duration is 10-22 weeks to final follow up visit.

# 2. Rationale and background

## 2.1. Rationale

Sargramostim (Leukine®) is a yeast-derived recombinant humanized granulocyte-macrophage colony stimulating factor (rhuGM-CSF, sargramostim) and the only FDA approved GM-CSF (Leucine Package Insert). GM-CSF, a pleiotropic cytokine, is an important leukocyte growth factor known to play a key role in haematopoiesis, effecting the growth and maturation of multiple cell lineages as well as the functional activities of these cells in antigen presentation and cell mediated immunity (*1*). Since its initial FDA approval in 1991, over 500,000 patients have received Leukine®, providing extensive clinical and post-marketing data in a broad range of treated individuals - from preterm neonates to the elderly and including males and females - representing a well-characterized safety profile for Leukine®. Leukine® administered as a subcutaneous or intravenous injection is approved for six indications including use as a medical countermeasure for radiation exposure. The US Government currently holds Leukine® in the Strategic National Stockpile. Leukine® may benefit patients with beginning signs of Acute Respiratory Distress Syndrome (ARDS) due to COVID-19 Infection. GM-CSF is a critical cytokine for the health of lungs. The alveolar macrophages are dependent on GM-CSF for differentiation and normal functioning. In addition, GM-CSF is an immunomodulator that plays a critical role in host defense by promoting differentiation of dendritic cells, and stimulating antiviral immunity (*2-4*).

As described in detail below, it is being studied as an adjuvant therapy in the management of life-threatening infections to boost the hosts innate immune response to fight infection, reduce the risk of secondary infection, and in varied conditions to prevent infection during critical illness (*5-8*). In addition, it has been studied in pulmonary conditions that affect alveolar macrophages, such as autoimmune pulmonary alveolar proteinosis (“aPAP”), with beneficial outcomes (*9, 10*). We propose based on preclinical and clinical data and the safety data from more than 500,000 adult and pediatric patients treated with Leukine®, that patients with beginning signs of acute lung injury and/or ARDS due to COVID-19 infection be given Leukine®. ARDS due to COVID-19 carries a high mortality rate (*11*) and Leukine® may confer benefit by both active management of this complication as well as in prevention of secondary infections.

In animal models of postviral ARDS and mortality, GM-CSF has demonstrated immunomodulatory effects that improve the clinical response and symptoms associated with influenza and other viral respiratory infections (*12-14*), and represents a promising candidate for the prevention of ARDS in patients with COVID-19.

**Practical Advantages of LEUKINE® for Acute Respiratory Distress due to COVID-19**

| Advantage | Details |
|---|---|
| **Approved** for use by the FDA | - 6 indications |
| **Safe** in Pediatric and Adult populations, including elderly | - Established safety profile in pediatric and adult populations, including elderly (>500,000 patients) |
| **Available** for use in the SNS plus commercial distribution | - Current approvals provide a pre-existing distribution and stockpile resource<br>Existing formulation safe for use with Pari LC Sprint Jet and Vectura Akita 2 nebulizers<br>- US based manufacturing of BDS and FDP |
| **Proven** effective in fighting infection/enhancing immune response | - GM-CSF modulates immune response to influenza virus and promotes viral clearance<br>- GM-CSF activates dendritic cells and T-cells<br>- GM-CSF improves epithelial lung repair |

## 2.2. Background

The proposed development plan was guided by three specific considerations:

**1. Supportive Scientific Rationale:**
The biology and effects of GM-CSF on the lung, specifically alveolar macrophages and epithelial cells, as well its immunomodulatory activities in stimulating antiviral immunity make GM-CSF a critical cytokine for healthy pulmonary function and defence. Detailed studies have shown that GM-CSF is necessary for the maturation of alveolar macrophages from fetal monocytes and the maintenance of these cells in adulthood (*1*).
GM-CSF has a wide array of effects on myeloid cells. GM-CSF has been shown to be a myelopoietic growth factor that has pleiotropic effects not only in promoting the differentiation of immature precursors into polymorphonuclear neutrophils, monocytes/ macrophages and dendritic cells, but also in controlling the function of fully mature myeloid cells (*15*). GM-CSF is also known to reverse immunoparalysis seen in sepsis by immune activation, resulting in beneficial outcomes (*5*).

There is a large body of evidence generated with GM-CSF in animal studies suggesting the potential use in ARDS and infections (*16*). For the purpose of brevity, we will point to the data that reflects the potential value in viral lung infections and preventing secondary bacterial infections and progression to ARDS:

Halstead and colleagues demonstrated that in vivo high airway levels of GM-CSF profoundly rescue mice from lethal influenza pneumonia. While in vitro GM-CSF is canonically described as an M1-polarizing cytokine, their data demonstrated that in vivo, during influenza A virus infection, GM-CSF instead temporizes the type II interferon-induced M1 polarization of airway macrophages and reduces inflammation induced damage (*12, 13*). Unkel and colleagues demonstrated GM-CSF–dependent cross-talk between influenza virus infected alveolar epithelial cells and CD103+ dendritic cells is crucial for effective viral clearance and recovery from injury and thus pointing to the potential use of GM-CSF treatment in severe influenza virus pneumonia (*17*). Investigations have shown that GM-CSF conferred resistance to influenza in mice via alveolar phagocytes and through alveolar macrophages which became more resistant to influenza- induced apoptosis. Delivery of intranasal GM-CSF to wild-type mice also conferred resistance to influenza (*18*). There is evidence that inhaled GM-CSF prevents bacteremia in post influenza bacterial pneumonia primarily through locally-mediated improved lung antibacterial resistance to systemic bacteremia during influenza A viral infection (*13*).

*Conclusions:* GM-CSF confers resistance to influenza by enhancing innate immune mechanisms that depend on alveolar macrophages, which are dependent on GM-CSF for their health and normal functioning. Pulmonary delivery of this cytokine has the potential to reduce morbidity and mortality due to viral pneumonia. This is summarized in the diagram below:

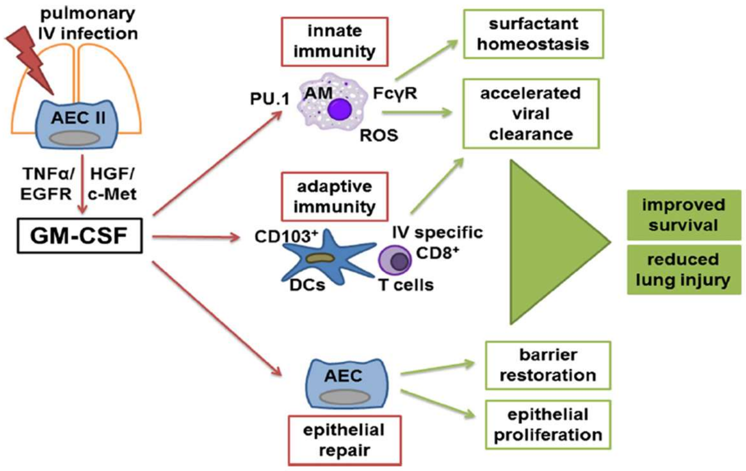


**Fig. 1** GM-CSF-modulated immune response to IV infection. After pulmonary IV infection GM-CSF is released from AEC II, mediated through HGF/c-Met and TGF-α/EGFR signaling. In an autocrine manner, it stimulates epithelial repair, including epithelial proliferation and barrier restoration. Innate and adaptive immunity are activated, resulting in accelerated viral clearance. Via PU.1, GM-CSF improves AM resistance, maturation, ROS production, and phagocytosis capacity, e.g., by the FcγR-mediated opsonophagocytosis. GM-CSF also stimulates activation and proliferation of DCs, especially CD103$^+$ DCs, and T cells and enhances Ag priming and IV-specific CD8$^+$ T cell recruitment. Altogether AEC GM-CSF leads to increased survival and reduced lung injury. *AEC* alveolar epithelial cells, *Ag* antigen, *AM* alveolar macrophage, *c-Met* hepatocyte growth factor receptor, *DC* dendritic cell, *EGFR* epithelial growth factor receptor, *FcγR* Fcɣ receptor, *GM-CSF* granulocyte and macrophage colony stimulating factor, *HGF* hepatocyte growth factor, *PU.1* transcription factor PU.1, *ROS* reactive oxygen species, *TGF-α* transcriptional growth factor α

**2. Experience: Use of Leukine® has beneficial effect in the treatment of conditions that are similar to ARDS seen with COVID-19.**

A small (18 patient) double blind randomized placebo controlled clinical trial of low-dose (3mcg/kg daily for 5 days) intravenous GM-CSF treatment in adult patients with severe sepsis and respiratory dysfunction, led to the conclusion that GM-CSF treatment was associated with improved gas exchange and might play a homeostatic role (*6*). In a phase II study, 130 patients with severe sepsis with respiratory dysfunction were randomized to GM-CSF (250mcg/m$^2$ intravenously daily for 14days) or placebo. The results showed an improvement in 28day mortality on GM-CSF; this did not reach statistical significance due to the small sample size (*7*).

Herold and colleagues used Leukine® by inhalation route on a compassionate basis in six patients with moderate to severe community-acquired pneumonia or ventilator-associated pneumonia ARDS who were not improving despite all measures and at least 6 days of mechanical ventilation(*8*). 125mcg of Leukine® were applied by Aeroneb Solo device (Covidien, Neustadt, Germany) at an interval of 48 hours. Compared to historical controls, the authors observed significant improvement in oxygenation and lung compliance with GM-CSF therapy. This resulted in improved morbidity using standard scoring systems and 4 of the six patients recovered and were discharged from the hospital. There is an ongoing study of inhaled GM-CSF across multiple centers in Germany (GI HOPE; NCT02595060) recruiting patients with diagnosis of pneumonia associated ARDS.

There is a large body of evidence of inhaled Leukine® in autoimmune pulmonary alveolar proteinosis (aPAP), which results in accumulation of surfactant in alveolar sacs with resultant hypoxia. Tazawa and colleagues conducted a phase II study of inhaled Leukine® at 9 pulmonary centers throughout Japan in patients with unremitting or progressive aPAP with hypoxia and symptoms (*9*). Patients received 250mcg daily by inhalation, using an LC-PLUS nebulizer with a manual interrupter valve connected to a PARI Turbo BOY compressor, for 7 days and this cycle was repeated every other week for six cycles (total 12 weeks). The treatment was well tolerated with no serious adverse events. Adverse events were reported in just 7 of the 39 patients oxygenation, radiological changes as well as symptoms. Following these results, a larger randomized phase 3 study (PAGE study) was conducted by the Japanese investigators in 12 centers. 64 patients with mild to moderate aPAP with hypoxia were randomized to receive placebo or Leukine® (33 patients) at a dose of 125mcg twice a day for 7days

followed by a week of no treatment. This two-week cycle was repeated 12 times over a period of 24 weeks. The treatment was again well tolerated with no significant differences in adverse events between the two groups. The GM-CSF treated patients had significantly improved hypoxia parameters and radiographic changes (*10*). This clinical experience of use of Leukine® in viral pneumonia suggests salutary effects. In addition, these studies establish the safety of inhaled Leukine® and provide evidence for activity of inhaled Leukine®.

**3. Expediency:** Toxicology, pharmacologic and safety data supports the immediate clinical use of Leukine® in hypoxic respiratory failure with acute lung injury leading to ARDS due to COVID-19. Investigator brochure is available and contains detailed information on toxicity.

## 2.3. Risk/Benefit Assessment

COVID-19 poses a very significant risk of mortality of 3-7% and this percentage rises to mortality of 20% in patients with co-morbidity (*11, 19*). Of all infected patients, some 15-20% develop severe respiratory symptoms necessitating hospital admission. Around 5% of infected patients will require invasive mechanical ventilation, and many of those (40-50% will die). The current world-wide pandemic of COVID-19 is putting unforeseen stress on the entire primary, secondary and tertiary medical system, leading to unseen triage of patients that potentially benefit or not from admission to ICU units when they develop respiratory failure.

GM-CSF (sargramostim, Leukine®) has been given systemically to almost 500.000 patients in the past. It is therefore a well characterized product. Inhalation of GM-CSF has also been used to treat patients with interstitial lung disease and reduced oxygen saturation (i.e. partial acute hypoxic respiratory failure) with few significant side effects above the placebo arm. The protocol is set up to give twice daily inhalation with GM-CSF, followed by intravenous administration if the patient would move to the ICU unit on mechanical ventilation.

Although GM-CSF has been given systemically and via inhalation to patients with pneumonia-associated ARDS, there are no current data on the safety profile of this drug in patients with COVID-19. Given the severity of the clinical syndrome caused by COVID-19, and the prior triage of patients before hospital admission to the COVID-19 ward, this trial will be performed in a hospital setting on a COVID-19 ward with close monitoring of vital parameters (continuous ECG, oxygen saturation, temperature, vital clinical signs), which will allow intermediate intervention should serious side effects occur. Once on the ICU unit, patients will be intensively monitored for all vital parameters, as part of the routine ICU monitoring.

There are currently no treatments directed at improving lung repair and local immunity in COVID-19 patients, and no treatment that attempt to halt the progression from manageable acute hypoxic respiratory failure to ARDS. Preventing such progression to ARDS could have a huge impact on the foreseeable overflow of the ICU units. We therefore believe the benefits of administering inhaled GM-CSF treatment in early stage COVID-19 acute hypoxic respiratory failure outweighs the risks associated with a phase 4 IMP administration via a different route and unknown indication.

## 2.4. Limitations

There is a large number of COVID-19 infected patients that are currently being hospitalized across the globe. In just 9 days time, our COVID-19 ward at Ghent University Hospital has admitted 25 confirmed cases, of which a significant portion (50%) already fulfill eligibility criteria for the current proposed protocol. We therefore believe that given the current ascending part of the epidemiology curve, with numbers of patients rising sharply, there will be no shortage of patients that are eligible.
Partner Therapeutics has offered to give (free of charge) enough GM-CSF to treat 20 patients for a 10 day period and an additional 20 controls for 5 days (should deterioration occur). There are large

amounts of GM-CSF in the United States strategic national stockpile, so should this therapy work, there might be immediate worldwide application of a GM-CSF inhalation therapy.

## 3. Objectives

### 3.1. Primary Objectives

This is phase 4 academic, prospective, randomized, open-label, interventional study designed to investigate the efficacy of sargramostim (Leukine®) in improving oxygenation and short- and long-term outcome of COVID-19 patients with acute hypoxic respiratory failure. There are currently no treatments directed at improving lung repair and local immunity in COVID-19 patients, and no treatment that attempt to halt the progression from manageable acute hypoxic respiratory failure to ARDS in patients with COVID-19 infection.

**Justification for our objective** is that preventing progression from early acute hypoxic respiratory failure to ARDS could have a huge impact on the foreseeable overflow of the ICU units, that is already happening in some countries and is bound to happen on a global scale. The outcome of our study could thus have large impact from a medical, ethical and economic perspective.

The **hypothesis of the proposed intervention** is that GM-CSF has profound effects on antiviral immunity, can provide the stimulus to restore immune homeostasis in the lung with acute lung injury post COVID-19, and can promote lung repair mechanisms, that lead to a 25% improvement in lung oxygenation parameters.

This hypothesis is based on experiments performed in mice showing that GM-CSF treatment can prevent mortality and prevent ARDS in mice with post-viral acute lung injury.

**To address our hypothesis**, we will randomize patients with confirmed COVID-19 with acute hypoxic respiratory failure (saturation below 93% on minimal 2 l/min O2 or PaO2/FiO2 <350) to receive sargramostim 125mcg twice daily for 5 days as a nebulized inhalation on top of standard of care (active group), or to receive standard of care treatment (control group). Upon progression of disease requiring initiation of non-invasive or invasive mechanical ventilatory support within the 5 day period, in patients in the active group, inhaled sargramostim will be replaced by intravenous sargramostim 125mcg/m$^2$ body surface area once daily until the 5 day period is reached.

**To measure the effectiveness** of sargramostim on restoring lung homeostasis, the **primary endpoint** of this intervention is **measuring oxygenation** after 5 DAYS of inhaled (and intravenous) treatment through assessment of pretreatment and post-treatment ratio of PaO2/FiO2 and through measurement of the P(A-a)O2 gradient, which can easily be performed in the setting of clinical observation of patients admitted to the COVID -19 ward or ICU COVID-19 unit. During the 5 day treatment period, we will perform daily measurements of oxygen saturation (pulse oximetry) in relation to FiO2, and the slope of alterations in these parameters could also be an indicator that our hypothesis is correct.

Comparison will be between active group A receiving sargramostim on top of standard of care and control group B receiving standard of care.

Data from the Wuhan COVID-19 epidemic show that patients that deteriorate are facing a prolonged period of mechanical ventilation. Therefore, from day 6 onwards, progressive patients in the active group will have the option to receive an additional 5 days of IV sargramostim, based on the treating physician's assessment. This group will be called group C. In the control group, for patients with

progressive disease requiring (non)-invasive mechanical ventilatory support, from day 6 onwards, the treating physician will have the option to initiate IV sargramostim 125mcg/$m^2$ body surface area once daily for 5 days. This group will be called group D. Comparisons of Group A (early 5 day intervention with sargramostim) with Group D (late 5 day intervention with sargramostim) will also be very informative.

### 3.2. Secondary Objectives

**- to study if early intervention with sargramostim is safe (number of AEs/SAEs)**

- **to study if early intervention with inhaled sargramostim affects clinical outcome defined by duration of hospital stay,**
**Mean change in 6-point ordinal scale between day 1 and day 6**
**Mean change in clinical sign score between day 1 and day 6**
**Time to clinical sign score <6 maintained for 24h**
**Mean change of SOFA score between day 1 and day 6 or between day 1 and day 11.**
**Mean change NEWS2 score score between day 1 and day 6 or between day 1 and day 11.**
**Tine to NEWS2 score less than 2 for at least 24h**

- **to study if early intervention with sargramostim affects the rate of nosocomial infection**

**- to study if early intervention with inhaled sargramostim affects progression to mechanical ventilation and/or ARDS**

-**to study if treatment with sargramostim affects all cause mortality rate at 20 weeks post inclusion**

-**to study if treatment with sargramostim affects features of secondary haemophagocytic lymphohistiocytosis**, **as defined by Hs score (temp, organomegaly, cytopenia, triglycerides, fibrinogen, ferritin, AST and known immunosuppression)**

- **to study if treatment with sargramostim has a favourable effect on long term 10-20 week follow up**

## 4. End Points + Time Points

### 4.1. Primary End Points + Time Points

**To measure the effectiveness** of sargramostim on restoring lung homeostasis, the **primary endpoint** of this intervention is **measuring oxygenation** after 5 DAYS of inhaled (and intravenous) treatment through assessment of pretreatment (day 0) and post-treatment (day 5) ratio of PaO2/FiO2 and through measurement of the P(A-a)O2 gradient, which can easily be performed in the setting of clinical observation of patients admitted to the COVID -19 ward or ICU COVID-19 unit. Preferentially, this measurement should be done in the upright position, while breathing room air for a minimum of 3 minutes.. If this is impossible due to need for supplemental oxygen, FiO2 and oxygen supplementation method should be recorded in patient record, so that A-a gradient can be normalized for age expected normal A-a gradient while on supplemental oxygen use.
During the 5 day treatment period, we will perform daily measurements of oxygen saturation (pulse oximetry) in relation to FiO2, and the slope of alterations in this parameters could also be an indicator that our hypothesis is correct.
If the patient leaves hospital prior to the day 6 analysis point, oxygenation at day of discharge will be used as value for measuring primary endpoint.

## 4.2. Secondary End Points + Time Points

**- to study if early intervention with sargramostim is safe (number of AEs/SAEs)**
Although sargramostim has been given previously by inhalation to patients with ARDS and interstitial lung disease, data on safety in patients with COVID-19 infection are currently lacking. Since we are randomizing against 5 days of no sargramostim treatment, comparison of AEs and SAEs between group A and group B will be very informative.

-**to study if early intervention with inhaled sargramostim affects clinical outcome**
defined by length of hospital stay
mean change in 6-point ordinal scale change between day 1, day 6 and

**- to study if early intervention with sargramostim affects the rate of nosocomial infection**
Patients with viral respiratory infection are at risk of secondary bacterial infections. As part of routine clinical care, sputum samples will be collected in patients suspected of secondary bacterial pneumonia, and checked for the presence of bacteria.

**-to study if early intervention with inhaled sargramostim affects progression to mechanical ventilation and/or ARDS**
Decreasing oxygenation often leads to the need for non-invasive or invasive mechanical ventilation, and if severe enough to a diagnosis of ARDS. We will therefore as a secondary endpoint also study if early intervention with inhaled sargramostim prevents progression to criteria-defined ARDS (according to the American-European Consensus Conference (AECC) diagnostic criteria for ARDS: acute onset; ratio of partial pressure of arterial oxygen to fraction of inspired oxygen ($PaO_2/FiO_2$) of 200 or less, regardless of positive end-expiratory pressure; bilateral infiltrates seen on frontal chest radiograph; and pulmonary artery wedge pressure of 18 mm Hg or less when measured, or no clinical evidence of left atrial hypertension), requiring high-flow oxygen devices, non-invasive mechanical ventilation, mechanical ventilation, by measuring the day from admission when this diagnosis is made or therapies are initiated.

**-to study if treatment with sargramostim affects all-cause mortality rate at 4 and 20 weeks post inclusion.**

**-to study if treatment with sargramostim affects features of secondary haemophagocytic lymphohistiocytosis**.
A large subset of patients with severe COVID-19 developing respiratory failure might have a cytokine storm syndrome, designated as secondary haemophagocytic lymphohistiocytosis (sHLH). sHLH is an under-recognised, hyperinflammatory syndrome characterised by a fulminant and fatal hypercytokinemia with multi-organ failure. Cardinal features of sHLH include unremitting fever, cytopenias, and hyperferritinaemia; hypertriglyceridemia, pulmonary involvement can present as ARDS. A cytokine profile resembling sHLH is associated with COVID-19 disease severity, characterised by increased interleukin (IL)-2, IL-7, granulocyte-colony stimulating factor, interferon-γ inducible protein 10, monocyte chemoattractant protein 1, macrophage inflammatory protein 1-α, and tumour necrosis factor-α.
Predictors of fatality from a recent retrospective, multicentre study of 150 confirmed COVID-19 cases in Wuhan, China, included elevated ferritin (mean 1297·6 ng/ml in non-survivors vs 614·0 ng/ml in survivors; p<0·001) and IL-6 (p<0·0001), suggesting that mortality might be due to virally driven hyperinflammation.

To address the effect of sargramostim treatment on sHLH, we will measure levels of ferritin, these chemokines and cytokines at the beginning of the trial day 0 and after the initial 5 day treatment. PBO including leukocytes and lymphocytes are performed on a routine clinical basis in these patients.

- **to study if treatment with sargramostim has a favourable effect on long term 10-20 week follow up**

At 10-20 weeks after discharge from hospital, patients will be seen on routine check-up by pulmonologist, who will perform a clinical exam, pulmonary function tests (including FVC, TLC and diffusion capacity), and a laboratory (ferritin, lymphocytes, leukocytes).

# 5. Study design

## 5.1. Description of study design

This is phase 4 academic, prospective, randomized, open-label, interventional study designed to investigate the efficacy of sargramostim (Leukine®) in improving oxygenation and short- and long-term outcome of COVID-19 patients with acute hypoxic respiratory failure. There are currently no treatments directed at improving lung repair and local immunity in COVID-19 patients, and no treatment that attempt to halt the progression from manageable acute hypoxic respiratory failure to ARDS in patients with COVID-19 infection. **Justification for our objective** is that preventing progression from early acute hypoxic respiratory failure to ARDS could have a huge impact on the foreseeable overflow of the ICU units, that is already happening in some countries and is bound to happen on a global scale.

The **hypothesis of the proposed intervention** is that GM-CSF has profound effects on antiviral immunity, can provide the stimulus to restore immune homeostasis in the lung with acute lung injury post COVID-19, and can promote lung repair mechanisms, that lead to a 25% improvement in lung oxygenation parameters. This hypothesis is based on experiments performed in mice showing that GM-CSF treatment can prevent mortality and prevent ARDS in mice with post-viral acute lung injury.

We will randomize patients with confirmed COVID19 with acute hypoxic respiratory failure (saturation below 93% on minimal 2 l/min O2 or PaO2/FiO2 <350) to receive sargramostim 125mcg twice daily for 5 days as a nebulized inhalation on top of standard of care (active group), or to receive standard of care treatment (control group). Upon progression of disease to requiring invasive mechanical ventilatory support within the 5 day period, in patients in the active group, inhaled sargramostim will be replaced by intravenous sargramostim 125mcg/$m^2$ body surface area until the 5 day period is reached.
Dosing of inhaled and systemic sargramostim are based on prior experience of this drug in patients with pulmonary alveolar proteinosis (inhaled) and with pneumonia associated ARDS (inhaled and intravenous). The inhaled route is preferred first, because high local concentrations of GM-CSF have a favourable effect on lung immunity, lung homeostasis and lung repair. The switch to intravenous treatment with deterioration requiring initiation of mechanical ventilation is necessitated by the fact that patients with COVID-19 poorly tolerate ventilation in the absence of high level positive end expiratory pressure (PEEP), especially when they develop ARDS. For giving the sargramostim via inhalator in a ventilated patient, this would involve PEEP-free ventilation for at least 10-15 minutes, which will not be tolerated in COVID-19 associated severe hypoxic respiratory failure and/or ARDS according to expert opinion (Prof. Dr. Pieter Depuydt, Intensive Care Unit, UZ Ghent).

**To measure the effectiveness** of sargramostim on restoring lung homeostasis, the **primary endpoint** of this intervention is **measuring oxygenation** after 5 days of inhaled (and intravenous) treatment through assessment of pretreatment and post-treatment ratio of $PaO2/FiO_2$ and through measurement of the $P(A\text{-}a)O_2$ gradient, which can easily be performed in the setting of clinical observation of patients admitted to the COVID -19 ward or ICU COVID-19 unit. Supplemental oxygen use will be recorded, and if needed A-a gradient will be normalized against expected age- and supplemental oxygen dependent A-a gradient. During the 5 day treatment period, we will perform daily measurements of oxygen saturation (pulse oximetry) in relation to FiO2, and the slope of alterations in this parameters could also be an indicator that our hypothesis is correct. If the patient leaves hospital prior to the day 6 analysis point, oxygenation at day of discharge will be used as value for measuring primary endpoint.

Comparison will be between active group A receiving sargramostim on top of standard of care and control group B receiving standard of care.

Data from the Wuhan COVID-19 epidemic show that patients that deteriorate are facing a prolonged period of mechanical ventilation. Therefore, from day 6 onwards, progressive patients in the active group will have the option to receive an additional 5 days of IV sargramostim, based on the treating physician's assessment. This group will be called group C. In the control group with progressive disease requiring invasive mechanical ventilatory support or developing ARDS, from day 6 onwards, the treating physician will have the option to initiate IV sargramostim 125mcg/$m^2$ body surface area once daily for 5 days. This group will be called group D. Comparisons of Group A (early 5 day intervention with sargramostim) with Group D (late 5 day intervention with sargramostim) will also be very informative.

## 5.2. End of Study Definition

### 5.2.1. For an individual subject

The subject has completed the study if he or she has completed all phases of the study, including the last visit (week 10-20 clinical follow up visit) or the last scheduled procedures, as described in this protocol (see section "9. Study Specific Procedures").

### 5.2.2. For the whole study

Overall, the end of the study is reached when the last study procedure for the last subject has occurred: last subject, last visit (LSLV).
As soon as the whole study has ended (cfr. the definition above), the co-ordinating Investigator shall notify the HIRUZ Clinical Trial Unit, so that the Competent Authority and the Ethics Committee can be informed in a timely manner according to the regulatory requirements (within 90 days after end of the study, or if the study had to be terminated early, this period must be reduced to 15 days and the reasons should clearly explained).

## 5.3. Estimated duration of the study

There is a large number of COVID-19 infected patients that are currently being hospitalized across the globe. In just 9 days time, our COVID-19 ward at Ghent University Hospital has admitted 25 confirmed cases, of which a significant portion (50%) already fulfill eligibility criteria for the current proposed protocol. We therefore believe that given the current ascending part of the epidemiology curve, with numbers of patients rising sharply, there will be no shortage of patients that are eligible. We estimate the study to terminate in 30 weeks, including last clinical follow up visits.

# 6. Inclusion and Exclusion Criteria

## 6.1. Inclusion Criteria

The following patients will be enrolled

Recent (≤2weeks prior to randomization) - Confident COVID-19 diagnosis confirmed by antigen detection test and/or PCR and/or positive serology, or any emerging and validated diagnostic laboratory test for COVID-19 within this period.

-In some patients, it may be impossible to get a confident laboratory confirmation of COVID-19 diagnosis after 24h of hospital admission because viral load is low and/or problems with diagnostic sensitivity. In those cases, in absence of an alternative diagnosis, and with highly suspect bilateral ground glass opacities on recent (<24h) chest-CT scan (confirmed by a radiologist and pulmonary physician as probable COVID-19), a patient can be enrolled as probable COVID-19 infected. In all cases, this needs confirmation by later seroconversion.
-Presence of acute hypoxic respiratory failure defined as (either or both)
  saturation below 93% on minimal 2 l/min O2
  PaO2/FiO2 below 350
-Admitted to specialized COVID-19 ward
-Age 18-80
-Male or Female
-Willing to provide informed consent

## 6.2. Exclusion Criteria

-Patients with known history of serious allergic reactions, including anaphylaxis, to human granulocyte-macrophage colony stimulating factor such as sargramostim, yeast-derived products, or any component of the product.

-mechanical ventilation before start of study

-Patients enrolled in another investigational drug study

-Pregnant or breastfeeding females (all female subjects regardless of childbearing potential status must have negative pregnancy test at screening)

- patients with peripheral white blood cell count above 25.000 per microliter and/or active myeloid malignancy

-patients on high dose systemic steroids (> 20 mg methylprednisolone or equivalent)

-patients on lithium carbonate therapy

-Patients with serum ferritin >2000 mcg/ml (which will exclude ongoing HLH)

### 6.2.1. Screen failures

Screen failures are defined as subjects who consent to participate in the clinical study but are not subsequently randomly assigned to the study intervention or entered in the study. A minimal set of screen failure information will be kept to ensure transparent reporting of screen failure subjects.

# 7. Target Population

## 7.1. Subjects

### 7.1.1. Number of subjects and planned recruitment rate

There is a large number of COVID-19 infected patients that are currently being hospitalized across the globe. In just 9 days time, our COVID-19 ward at Ghent University Hospital has admitted 25 confirmed cases, of which a significant portion (50%) already fulfill eligibility criteria for the current proposed protocol. Similar numbers of patients are currently being seen in all centers.. We therefore believe that given the current ascending part of the epidemiology curve, with numbers of patients rising sharply, there will be no shortage of patients that are eligible.

The number of subjects that will be included in this study is: 80.
These are divided into following sub-groups:

**Group A** : active **sargramostim** treatment group, treatment for initial 5 days, no deterioration after 5 days
Number of patients : 40

**Group B** : control group : no treatment with sargramostim in first 5 days
Number of patients : 40

**Group C and D** :
Data from the Wuhan COVID-19 epidemic show that patients that deteriorate are facing a prolonged period of mechanical ventilation. Therefore, from day 6 onwards, progressive patients in the active group will have the option to receive an additional 5 days of IV sargramostim sargramostim 125mcg/m2 body surface area once daily, based on the treating physician's assessment. This group will be called group C. It is estimated that some 30% of patients might deteriorate and require non-invasive or invasive mechanical ventilation, giving potentially rise to 12 patients that progress from group A to group C, if the clinician decides to move forward with the drug.

In the control group progressing to requiring invasive or non-invasive mechanical ventilatory support, from day 6 onwards, the treating physician will have the option to initiate IV sargramostim 125mcg/m$^2$ body surface area once daily for 5 days. This group will be called group D. It is estimated that some 30% of patients might deteriorate to mechanical ventilation or ARDS, giving potentially rise to 12 patients that progress from group A to group C, if the clinician decides to move forward with the drug

Comparisons between group A (early sargramostim) versus group B (no sargramostim) at day 6 will be important for reaching primary endpoint, and for key secondary endpoints. Comparisons of Group A (early 5 day intervention with sargramostim) with Group D (late 5 day intervention with sargramostim) will also be very informative for secondary endpoint analysis.

### 7.1.2. Withdrawal and replacement of subjects

Subjects are free to withdraw from participation in the study at any time upon request.

An investigator may discontinue or withdraw a subject from the study for the following reasons:

- allergic reactions (anaphylactic shock) to sargramostim
- Pregnancy
- Progression to non-invasive or invasive mechanical ventilation and/or ARDS between screening and randomization
- Significant study intervention non-compliance
- If any clinical adverse event (AE), laboratory abnormality, or other medical condition or situation occurs such that continued participation in the study would not be in the best interest of the subject
- If the subject meets an exclusion criterion (either newly developed or not previously recognized) that precludes further study participation

In all cases, the reason why subjects are withdrawn must be recorded in detail in the eCRF and in the subject's medical records.

If a patient decides to leave hospital before day 6 of the study, for example because of clinical improvement, the oxygenation parameters at day of discharge will be used to calculate the primary endpoint measurement.

The following actions must be taken if a subject fails to return to the clinic for a required study visit (visit at 10-20 weeks post end of study) :

- The site will attempt to contact the subject and reschedule the missed visit within 4 weeks and counsel the subject on the importance of maintaining the assigned visit schedule and ascertain if the subject wishes to and/or should continue in the study.
- Before a subject is deemed lost to follow-up, the investigator or designee will make every effort to regain contact with the subject (where possible, 3 telephone calls and, if necessary, a certified letter to the subject's last known mailing address or local equivalent methods). These contact attempts should be documented in the subject's medical record or study file.
- Should the subject continue to be unreachable, he or she will be considered to have withdrawn from the study with a primary reason of lost to follow-up.

## 7.2. Method of recruitment

Subjects will be recruited at the COVID-19 hospitalization ward at the participating centers. The study will be proposed by the treating physician to all subjects with PCR-Confirmed COVID-19 infection and a presence of acute hypoxic respiratory failure.

There will be no compensation for study participation. Partner Therapeutics Inc. is providing sargramostim to the study subjects, free of charge.

Since this is a hospital based trial, taking place over a minimum of five days in which patients are severely ill, we suspect the retention in the trial to be high.

## 7.3. Screening

Patients will be informed about the study by the treating physician.

After receiving full explanation, having received sufficient time to considerer the trial, asking questions and receiving satisfying responses to all questions, patients will be asked to sign ICF.

A serum pregnancy test will be done (female patients only).
Medical history will be checked for review of exclusion criteria and relevant subject information.

Patients will be continuously monitored on the COVID-19 ward.
Exams (standard of care) include, but are not limited to:

- ECG
- Chest X-Ray, and CT-scan
- Laboratory tests for leukocyte formula, kidney and liver function, ferritin levels
- Vital signs
- Pulse oximetry, Arterial blood gas, capnography

As soon as all in- and exclusion criteria are checked and patient is considered eligible, patient can be randomized. There is no minimal window to randomize the patient.

# 8. Investigational Medicinal Product (IMP)

## 8.1. Name of the IMP

LEUKINE®

### 8.1.1. Composition and active substance of the IMP

Sargramostim, Granulocyte macrophage colony-stimulating factor (GM-CSF), is the active substance of Leukine®.

### 8.1.2. Producer and Distributor of the IMP

The producer and distributor of Leukine® is Partner Therapeutics Inc, an integrated commercial-stage biotech company focused on the development and commercialization of therapeutics that improve health outcomes in the treatment of cancer. The distribution of IMP will be done by Tanner Pharma.

### 8.1.3. Preparation + Dosage + administration of the IMP

For inhalation: LEUKINE® is a sterile, preservative-free lyophilized powder that requires reconstitution with 4mL normal saline solution, to reach a concentration of 62,5 mcg/ml. Once reconstituted, LEUKINE® can be inhaled as an aqueous aerosol using either a vibrating mesh nebulizer or jet nebulizer, aerosolizing 2 ml twice daily. Reconstituted LEUKINE® solution for inhalation should be used within 16 hours following reconstitution and/or dilution. Dosage for inhalation: 125mcg twice daily via nebulizer. Nebulizing is preferably done in an isolation negative pressure chamber, and if not, personnel should use an FFP2 mask. Patient should self-administer the medication and where possible, the room should not be entered within one hour after administration.

For intravenous injection: LEUKINE® injection in 0.9% Sodium Chloride Injection, USP. Dilute LEUKINE® for intravenous infusion in 0.9% Sodium Chloride Injection, USP. If the final concentration of LEUKINE®

is below 10 mcg/mL, add Albumin (Human) at a final concentration of 0.1% to the saline prior to addition of LEUKINE to prevent adsorption to the components of the drug delivery system. To obtain a final concentration of 0.1% Albumin (Human), add 1 mg Albumin (Human) per 1 mL 0.9% Sodium Chloride Injection, USP (e.g., use 1 mL 5% Albumin [Human] in 50 mL 0.9% Sodium Chloride Injection, USP). Once diluted for infusion, LEUKINE® is stable for 6h. Dosage for intravenous injection: 125mcg/m$^2$/day over a 4-hour period for up to 5 days.

### 8.1.4. Permitted dose adjustments and interruption of treatment

No dose adjustments and interruptions are permitted during this trial. In case of anaphylaxis or severe AE, the drug will be immediately interrupted.

### 8.1.5. Duration of treatment

LEUKINE® will be administered for 5 days, with a possible 5 day extension to a maximum of 10 days in case of progression of disease and need for mechanical ventilation.

### 8.1.6. Packaging and Labeling of the IMP

LEUKINE® (sargramostim) for injection is a sterile, preservative-free, white lyophilized powder supplied in a carton containing five 250 mcg single-dose vials.

LEUKINE® (sargramostim) injection is a sterile, clear, colorless solution preserved with 1.1% benzyl alcohol supplied in a carton containing one 500 mcg/mL multiple-dose vial and a carton containing five 500 mcg/mL multiple-dose vials (NDC 0024-5844-05).

Storage and Handling : Leukine should be stored at 4 °C.

Drug will be labeled by Pharmacy UZ Ghent (for UZ Ghent enrolment) for inhaled or intravenous use.

### 8.1.7. Storage conditions of the IMP

Store LEUKINE® vials refrigerated at 2°C-8°C (36°F-46°F) in the original carton to protect from light. Do not freeze or shake. Do not use beyond the expiration date printed on the vial.
Leukine® is to be shipped refrigerated at 2°C-8°C (36°F-46°F). The medication will be delivered to the pharmacy of the participating centers. Temperature during shipment and storage is to be monitored continuously. Whenever a temperature deviation occurs, Partner Therapeutics Inc. should be contacted. Partner Therapeutics Inc. might allow further use of the medication vials depending on the duration and intensity of the temperature excursion. The co-ordinating investigator should be informed of this deviation as well.

### 8.1.8. Known side effects of the medication

To date, there have been no new safety signals associated with LEUKINE® (sargramostim). Observed side effects with aerosolized LEUKINE® at 250mcg dose and in at least one evaluation have included: bronchospasm, cough, dyspnea, a decrease in vital capacity and/or forced expiratory volume associated with bilateral infiltrates, pleural effusions, increased phlegm, throat irritation, and back pain.

## 8.2. Concomitant / Rescue Medication

There are no restrictions regarding concomitant/rescue medication.

# 9. Study Specific Procedures

Patients will be informed about the study by the treating physician.
After receiving full explanation, having received sufficient time to considerer the trial, asking questions and receiving satisfying responses to all questions, patients will be asked to sign ICF.
The ICF process will be performed before any other study related procedure.

## 9.1. Randomisation

In this open label trial patients will be randomized in a 1:1 ratio. Randomization in Belgium will be done using REDCap (electronic IVRS system).

## 9.2. Study specific interventions

This is a hospital based intervention trial, in which patients with COVID-19 will be treated at least for 5 days with sargramostim. Patients with COVID-19 infection and respiratory failure are severely ill, and will require multiple daily clinical exams, blood sampling, vital parameter measurements, blood oxygenation measurements, and chest X-rays. These are all part of the clinical management plan of the patients, and data stored in the electronic patient file will be used as part of the assessment of efficacy and safety profile of sargramostim.

On screening, blood sample will be taken, preferentially during routine blood sampling, to determine exclusion criteria (pregnancy, high ferritin level).

On day 1, prior to sargramostim treatment in group A, and during the day in group B control patients, a tube of blood serum (5 ml) and an EDTA tube (10 ml) will be collected for measuring blood cytokine and chemokine levels, and activation of immune cells in selected centers. Also in each center, an arterial blood gas determination via arterial puncture will be taken. This sample should be taken in an upright position, while breathing room air for a minimum of 3 minutes.. If this is impossible due to dependency on supplemental oxygen, FiO2, oxygen flow rate, and method of oxygen delivery should be noted in the patient file. If arterial blood gas is taken within 24h before first dose administration, as described in point° the arterial blood gas of screening can be used as D1 value.

Estimating FiO2 for nasal canula, face mask or face mask + reservoir

| Method | $O_2$ flow (l/min) | Estimated FiO2 (%) |
|---|---|---|
| Nasel cannula | 1 | 24 |
| | 2 | 28 |
| | 3 | 32 |
| | 4 | 36 |
| | 5 | 40 |
| | 6 | 44 |
| Nasopharyngeal catheter | 4 | 40 |
| | 5 | 50 |
| | 6 | 60 |
| Face mask | 5 | 40 |
| | 6-7 | 50 |
| | 7-8 | 60 |
| Face mask with reservoir | 6 | 60 |
| | 7 | 70 |
| | 8 | 80 |
| | 9 | 90 |
| | 10 | 95 |

On day 6 or on day of discharge before day 6 , a tube of blood serum (5ml) and an EDTA tube (10 ml) will be collected for measuring blood cytokine and chemokine levels, and activation of immune cells in selected centers. Also in each center, an arterial blood gas determination via arterial puncture will be taken.

On days 1-5, patients in group A will inhale sargramostim 125mcg twice daily for 5 days as a nebulized inhalation using a Philips InnoSpire Go portable mesh nebulizer on top of standard of care. This device is a handheld mesh nebulizer that can be fitted with a facial mask. Patients will be instructed prior to receiving the first dose on how to use this simple device, by a physician. This procedure is finished in 5-10 minutes, and will be performed twice daily, in the morning (between 6 a.m. and 11 a.m.) and evening (between 6 p.m. and 11 p.m.).

Upon progression of disease requiring initiation of invasive mechanical ventilatory support within the 5 day period, in patients in the active group, inhaled sargramostim will be replaced by intravenous sargramostim 125mcg/$m^2$ body surface area once daily until the 5 day period is reached. This administration will occur via a centrally placed catheter or peripheral catheter, that will be in place as part of routine medical care at the ICU.

On a final clinical visit between week 12-20 an additional serum tube (5ml) and an EDTA tube (10 ml) will be taken in selected centers.

## 9.3. Overview of collected data

1. patient demographics
    age, sex, ethnicity, day of admission
2. day of COVID-19 PCR positivity, and conversion to negative
2. patient biometry
    weight, length, BMI, body surface area
3. Clinical and laboratory parameters on screening day and during trial
    -first day of illness, potential source of infection
    -clinical examination findings (cyanosis, crepitation's and rales, heart murmurs, peripheral edema)
    -vital signs (temperature, blood pressure, heart rate, breathing rate)
    -pulse oximetry data (SaO2)
    -clinical blood gas sampling (PaO2, PaCO2, HCO3)
    -clinical chemistry sampling (ferritin, leukocyte formula, platelets, kidney and liver function, fibrinogen, triglycerides)
    -Chest X-ray and/or CT characteristics and radiology clinical report
    -in case of admission to ICU : invasive monitoring data (arterial blood pressure, PCWP, continuous O2 saturation, continuous ECG, ventilatory parameters (tidal volume, FiO2, PEEP pressure, peak pressure, minute ventilation)
4. All standard care drugs used during the trial and on day of enrolment of the trial, including oxygen flow rate.
5. Basic clinical data on prior medical history (prior lung diseases, smoking history, prior lung function measurements (preferentially within 5 preceding years), prior gas exchange measurements) and medication use will be collected from electronic medical record.
6. Study specific measurements

## 9.4. Schematic overview of the data collection & interventions

| **Procedures** | Screening | D1 | D5 | D6 (or discharge before day 6 ) | D10 | Follow-up (10-20 weeks after D1) |
|---|---|---|---|---|---|---|
| Informed consent | X | | | | | |
| Inclusion/exclusion criteria check | X | | | | | |
| Randomisation | | X | | | | |
| Medical History | X | | | | | |
| Lung function* | | | | | | X |
| Physical examination* | X | ---------------------------------------→ | | | | X |
| ECG* | X | ----------------------------→ | | | X | |
| Arterial blood gas° | | X§ | | X | | X |
| Chest X-ray* | X | ----------------------------→ | | | X | |
| Laboratory assessments* | X | ---------------------------------------→ | | | | X |
| Blood sampling (Selected centers only) | | X | | X | | X |
| Serum pregnancy test | X | | | | | |
| Vital signs (incl. height and weight)* | X | ---------------------------------------→ | | | | X |
| Pulse oximetry* | X | ---------------------------------------→ | | | | X |
| Device training with saline (if randomized in treatment group) | | X | | | | |
| IMP∞ | | (X) → | (X) | (X) → | (X) | |
| Drug Compliance | | | X | -----→ | X | |
| Concomitant medications | X | ---------------------------------------→ | | | | X |
| Adverse event check | X | ---------------------------------------→ | | | | X |

* Intervention is standard of care and is being performed regardless of inclusion in the study.
° This sample should be taken in an upright position, while breathing room air for a minimum of 3 minutes.. If this is impossible due to dependency on supplemental oxygen, FiO2, oxygen flow rate, and method of oxygen delivery should be noted in the patient file. [§]if arterial blood gas is taken within 24h before first dose administration, as described in point° the arterial blood gas of screening can be used as D1 value
∞ Patients randomized in the treatment group will receive inhaled sargramostim from D1 untill D5. In case of progression requiring mechanical ventilation within the first 5 days, IV sargramostim can be initiated until the 5 day period is reached. From day 6 onwards, progressive patients in the active group will have the option to receive an additional 5 days of IV sargramostim, based on the treating physician's assessment. Patients in the control group will have the option to receive 5 days of IV sargramostim in case of progression requiringmechanical ventilation, based on the treating physician's assessment.

Order of assessments:
IMP should always be administered after other assessments, where possible.

## 9.5. Restrictions for subjects during the study

There are no subject restrictions during this trial.

# 10. Sampling

## 10.1. Types and number of samples

In selected centers only:
D1: serum blood sample 5ml, EDTA blood sample, 10 ml
D6 or discharge before day 6 : serum blood sample 5ml, EDTA blood sample, 10 ml
W10-20 follow-up visit : serum blood sample 5ml, EDTA blood sample, 10 ml

## 10.2. Timepoints of sampling

These samples are to be taken on D1 and D6 (or discharge if before day 6) and on final follow up visit between week 10 and 20. There's no time window allowed.

## 10.3. Sample Handling & Analysis

In selected centers samples will be taken during hospitalization together with the blood draw for standard of care.

After clotting for 30-60minutes the samples will be processed at 1500RPM or 410 g during 10 minutes at room temperature.
3 aliquots will be filled and frozen at -80°C until further analysis.
Centrifugation and storage will be done by qualified personal.

EDTA blood samples will be processed to purify peripheral bloodmonocytes and stained for flow cytometric analysis of number of monocytes, HLA-DR expression on monocytes and dendritic cells, and lymphocyte activation.

Multiple cytokines and chemokines will be measured by multiplex bead based ELISA assay. Flow cytometry will be performed on paraformaldehyde fixed samples. Development of anti-drug antibodies (ADA) will be measured using protocol developed by Partner Therapeutics on serum samples taken at day 1 and follow up visit.

## 10.4. Sample Storage and/or shipping

Serum samples and frozen PBMCs will be stored at temperature monitored facilities of the participating research centres. .
Storage conditions: -80°C.

## 10.5. Future use of stored samples

Initially samples will be stored for the use as described within this protocol. If at a later time point samples will be stored for future use, they will be stored in FAGG certified biobank

# 11. Statistical Considerations

## 11.1. Sample size calculation

The outcome(s) on which the sample size calculation is based upon, is the primary endpoint measurement of oxygenation, defined as ratio of Pa02/FiO2 and P(A-a)$O_2$.

Sample calculation and power analysis have been performed using Genstat. The target difference is the difference measured at the primary endpoint (at day 5) between the control and the treated group. Given a sample size of 40 patients each, a minimal improvement of 10% in the treated group relative to the control group will be detected as significant at a significance level of 0,01 with a power of 0.90. The error variance was set at 100 units, corresponding with a standard deviation of 10 units.

## 11.2. Type of statistical methods

The statistical test to be used will be an F-test. A two-sample t-test is expected to give similar results.

## 11.3. Statistical analysis team

The statistical analysis will be performed by Gnomixx, a biostatistics consultancy company based in Ghent (Dr Marnik Vuylsteke). http://www.gnomixx.com/

# 12. Data handling

## 12.1. Method of data collection

Subjects that are included in the study , will be assigned a unique study number upon their registration in REDCap.. On all documents submitted to the coordinating center, sponsor or CI, patients will only be identified by their study number. The subject identification list will be safeguarded by the site. The name and any other directly identifying details will not be included in the study database.

### 12.1.1. Case Report Form

An electronic data capture (EDC) system, i.e. REDCap, will be used for data collection. Data reported on each eCRF should be consistent with the source data. If information is not known, this must be clearly indicated on the eCRF. All missing and ambiguous data will be clarified.
Only the data required by the protocol are captured in the eCRF. The eCRFs and the database will be developed, based on the protocol. The final eCRF design will be approved by the Co-ordinating Investigator.
All data entries and corrections will only be performed by study site staff, authorized by the investigator. Data will be checked by trained personnel (monitor, data manager) and any errors or inconsistencies will be clarified. The investigator must verify that all data entries in the eCRF are accurate and correct.

REDCap is provided and maintained by Vanderbilt University; a license for use was granted to the Health, Innovation and Research Institute (HIRUZ). REDCap is a web-based system.
The study site staff is responsible for data entry in REDCap.

#### 12.1.2. Data directly collected in the CRF (no source available)

*N*.A.

### 12.2. Data storage

The data is accessed through a web browser directly on the secure REDCap server. The server is hosted within the UZ Ghent campus and meets hospital level security and back-up requirements.

Privacy and data integrity between the user's browser and the server is provided by mandatory use of Transport Layer Security (TLS), and a server certificate issued by TERENA (Trans-European Research and Education Networking Association). All study sites will have access to REDCap. Site access is controlled with IP restriction.

### 12.3. Archiving of data

The investigator and sponsor specific essential documents will be retained for at least 25 years. At that moment, it will be judged whether it is necessary to retain them for a longer period, according to applicable regulatory or other requirement(s).

### 12.4. Access to data

Direct access will be granted to authorised representatives from the Sponsor, host institution and the regulatory authorities to permit study-related monitoring, audits and inspections.

Login in REDCap is password controlled. Each user will receive a personal login name and password and will have a specific role which has predefined restrictions on what is allowed in REDCap. Furthermore, users will only be able to see data of subjects of their own site. Any activity in the software is traced and transparent via the audit trail and log files.

# 13. Safety

## 13.1. Definitions

| Term | Definition |
|---|---|
| **Adverse Event (AE)** | Any untoward medical occurrence in a subject to whom a medicinal product has been administered, including occurrences which are not necessarily caused by or related to that product. |
| **Unexpected Adverse Event** | An adverse event, the nature or severity of which is not consistent with the applicable product information (e.g., Investigator's Brochure for an unapproved investigational product or package insert/summary of product characteristics for an approved product). |
| **Adverse Reaction (AR)** | An untoward and unintended response in a subject to an investigational medicinal product which is related to any dose administered to that subject.<br>The phrase "response to an investigational medicinal product" means that a causal relationship between a study medication and an AE is at least a reasonable possibility, i.e. the relationship cannot be ruled out.<br>All cases judged by either the reporting medically qualified professional or the Sponsor as having a reasonable suspected causal relationship to the study medication qualify as adverse reactions. |
| **Serious Adverse Event (SAE)** | A serious adverse event is any untoward medical occurrence that:<br>• results in death<br>• is life-threatening<br>• requires inpatient hospitalisation or prolongation of existing hospitalisation<br>• results in persistent or significant disability/incapacity<br>• consists of a congenital anomaly or birth defect<br>Other 'important medical events' may also be considered serious if they jeopardise the subject or require an intervention to prevent one of the above consequences.<br>NOTE: The term "life-threatening" in the definition of "serious" refers to an event in which the subject was at risk of death at the time of the event; it does not refer to an event which hypothetically might have caused death if it were more severe. |
| **Serious Adverse Reaction (SAR)** | An adverse event that is both serious and, in the opinion of the reporting Investigator, believed with reasonable probability to be due to one of the study treatments, based on the information provided. |
| **Suspected Unexpected Serious Adverse Reaction (SUSAR)** | A serious adverse reaction, the nature and severity of which is not consistent with the information about the medicinal product in question set out:<br>• in the case of a product with a marketing authorisation, in the summary of product characteristics (SmPC) for that product<br>• in the case of any other investigational medicinal product, in the investigator's brochure (IB) relating to the study in question |

<u>Attribution definitions</u>
An adverse event is considered associated with the use of the drug if the attribution is possible, probable or definitive.

*Not related*
An adverse event which is not related to the use of the drug.

*Unlikely*
An adverse event for which an alternative explanation is more likely - e.g. concomitant drug(s), concomitant disease(s), and/or the relationship in time suggests that a causal relationship is unlikely.

*Possible*
An adverse event which might be due to the use of the drug. An alternative explanation - e.g. concomitant drug(s), concomitant disease(s), - is inconclusive. The relationship in time is reasonable; therefore the causal relationship cannot be excluded.

*Probable*
An adverse event which might be due to the use of the drug. The relationship in time is suggestive (e.g. confirmed by dechallenge). An alternative explanation is less likely - e.g. concomitant drug(s), concomitant disease(s).

*Definitely*
An adverse event which is listed as a possible adverse reaction and cannot be reasonably explained by an alternative explanation - e.g. concomitant drug(s), concomitant disease(s). The relationship in time is very suggestive (e.g. it is confirmed by dechallenge and rechallenge).

## 13.2. Reporting requirements

### 13.2.1. AE reporting

AE's will be recorded from the first drug administration until the end of the study, as defined in section 5.2.
Special attention will be given to those subjects who have discontinued the study for an AE, or who experienced a severe or a serious AE. All AE's should be recorded in the patient's file and in the CRF.

### 13.2.2. SAE reporting

SAE's occurring during the entire study period will be reported as below.

All serious adverse events (initial and follow up information) and pregnancies occurring during this study must be reported by the local Principal Investigator within 24 hours after becoming aware of the SAE to:

- The local ethics committee (it is the responsibility of the local PI to report the local SAE's to the local EC)
- HIRUZ CTU of the University Hospital Ghent
- The National Coordinating Investigator (in case of multicenter studies)
  The company Partner Therapeutics that provides the IMP

This reporting is done by using the appropriate SAE form. For the contact details, see below.

#### 13.2.3. SUSAR reporting

In case the Coordinating Investigator, in consultation with HIRUZ CTU, decides the SAE is a SUSAR (Suspected Unexpected Serious Adverse Reaction), HIRUZ CTU will report the SUSAR to the Central EC and the FAMHP within the timelines as defined in national legislation. The Coordinating Investigator reports the SUSAR to all local PI's.
In case of a life-threatening and fatal SUSAR the entire reporting process must be completed within 7 calendar days. In case of a non life-threatening SUSAR the reporting process must be completed within 15 calendar days.

### 13.3. List of contact details for safety reporting

**HIRUZ CTU**:
Ghent University Hospital
C. Heymanslaan 10, 1K5
9000 Ghent, Belgium
e-mail: hiruz.ctu@uzgent.be
Tel: +32 9 332 05 00
Fax: +32 9 332 05 20

**Coordinating Investigator:**
Prof. dr. Bart Lambrecht
Ghent University Hospital
Department of pneumology
C. Heymanslaan 10, 1K5
9000 Ghent, Belgium
email: bart.lambrecht@ugent.be
Tel: +32 9 332 91 10

**Marketing Authorisation Holder:**
Partner Therapeutics,
Dr. Debasish Roychowdhury
e-mail: Debasish.Roychowdhury@partnertx.com
Tel: +16107721703

### 13.4. Flowchart Reporting

| *Type of Adverse Event* | *Action to be taken* |
|---|---|
| AE | List all AE's per subject in the patient's file and add this information to the CRF. |
| SAE | Notify to HIRUZ CTU within 24 hours after becoming aware of the SAE + add the SAE to a list that will be reported yearly (see section 13.8) |
| SAR | Notify to HIRUZ CTU within 24 hours after becoming aware of the SAE<br>→ HIRUZ CTU will submit to the central EC |

| | |
|---|---|
| | → study team informs company that provides the IMP |
| SUSAR | Notify to HIRUZ CTU within 24 hours after becoming aware of the SUSAR<br>→ HIRUZ CTU will submit to the central EC.<br>→ HIRUZ CTU will submit to the FAMHP<br>→ study team informs company that provides the IMP |

In case the (SU)SAR occurs at a local participating site, the local PI or study team should also contact:
- The local Ethics Committee
- The Co-ordinating Investigator

## 13.5. Events, excluded from reporting

COVID-19 infection is a very recent syndrome, on which few data are available. Normal symptoms and natural disease course symptoms that will not be reported as adverse events are dyspnea, coughing, malaise, fever, drop in oxygen saturation, progression to respiratory failure, progression to ARDS, severe drop in blood pressure in the ICU,progression to multi-organ failure.

## 13.6. Data Safety Monitoring Board (DSMB)

All study medication is registered and used in current practice. Despite the known safety profile of the study medications and study design, a DSMB is foreseen.

## 13.7. Development Safety Update Report

The Coordinating Investigator will provide DSURs once a year throughout the clinical study, or on request, to the Competent Authority (FAMHP in Belgium), Ethics Committee and Sponsor. This DSUR will include all SAE's (who were not categorized as SAR's and were not immediately reported to the EC).

The report will be submitted within 60 days after the start of the study, and will subsequently be submitted each year until the study is declared ended.

HIRUZ CTU can provide a template that can be used to complete this DSUR.

# 14. Monitoring/Auditing/Inspection

## 14.1. Monitoring

### 14.1.1. General

Monitoring of the study will be performed in compliance with GCP E6(R2) and the applicable regulatory requirements. The study team will be trained in an initiation visit by the monitor. A training and delegation log will be held. A detailed description of the monitoring tasks can be found in the latest version of the (study-specific) 'Monitoring plan'.

### 14.1.2. Monitoring team

Monitoring services will be provided by HIRUZ CTU. All relevant contact details (e.g. primary contact person, can be found in the 'Monitoring plan'.

### 14.1.3. Scope

Monitoring services will consist of the following (non-exhaustive list):
- review of informed consents and the followed process
- check on recruitment status
- checking for protocol deviations/violations
- checking GCP compatibility
- check on safety reporting compliance
- IMP handling and storage
- review of study data

…

## 14.2. Inspection

This study can be inspected at any time by regulatory agencies during or after completion of the study. Therefore access to all study records, including source documents, must be accessible to the inspection representatives. Subject privacy must be respected at all times, in accordance to GDPR, GCP and all other applicable local regulations.
The investigator/study team should immediately notify the sponsor if he or she has been contacted by a regulatory agency concerning an upcoming inspection.

## 14.3. Protocol Deviation policy

Sponsor and all investigators agree to take any reasonable actions to correct protocol deviations/violations noted during monitoring/inspection, in consultation with the monitoring team. All deviations must be documented on a protocol deviation log by the study team that is kept available at any time for monitoring/inspection purposes. Under emergency circumstances, deviations from the protocol to protect the rights, safety or well-being of human subjects may proceed without prior approval of the sponsor and the EC.

## 14.4. Serious breach to GCP and/or the protocol

Critical issues that significantly affect patient safety, data integrity and/or study conduct should be clearly documented and will be communicated with the Coordinating Investigator, HIRUZ CTU and possibly both the applicable Ethics Committee(s) and Competent authority. (Please contact HIRUZ CTU asap in case of a serious breach: hiruz.ctu@uzgent.be and/or +3293320500).
Early determination of the study (in a specific center or overall) may be necessary in case of major non-compliance.

# 15. Ethical and legal aspects

## 15.1. Good Clinical Practice

The study will be conducted cfr the latest version of the ICH E6 (R2) GCP guidelines, creating a standard for the design, conduct, performance, monitoring, auditing, recording, analyses and reporting of clinical studies that provides assurance that the data and reported results are accurate and that the rights, integrity and confidentiality of study subjects are protected.

## 15.2. Informed Consent

Eligible subjects may only be included in the study after providing written (witnessed, if needed) Ethics Committee-approved informed consent, or, if incapable of doing so, after such consent has been provided by a legally acceptable representative(s) of the subject.
Informed consent must be obtained before conducting any study-specific procedures (as described in this protocol).

Prior to entry in the study, the investigator must explain to potential subjects or their legal representatives the study and the implication of participation. Subjects will be informed that their participation is voluntary and that they may withdraw consent to participate at any time. Participating subjects will be told that their records may be accessed by competent authorities and by authorized persons without violating the confidentiality of the subject, to the extent permitted by the applicable law(s) and/or regulations. By signing the Informed Consent Form (ICF), the subjects or legally acceptable representatives are authorizing such access.
After this explanation and before entry to the study, written, dated and signed informed consent should be obtained from the subject or legally acceptable representative. The ICF should be provided in a language sufficiently understood by the subject. Subjects must be given the opportunity to ask questions.
The subject or legally acceptable representative will be given sufficient time to read the ICF and to ask additional questions. After this explanation and before entry to the study, consent should be appropriately recorded by means of either the subject's or his/her legal representative's dated signature or the signature of an independent witness who certifies the subject's consent in writing. After having obtained the consent, a copy of the ICF must be given to the subject.
In case the subject or legally acceptable representative is unable to read, an impartial witness must attest the informed consent.
Subjects who are unable to comprehend the information provided or pediatric subjects can only be enrolled after consent of a legally acceptable representative.
The following information should be added to the electronic patient dossier (EPD):

- which version of the ICF was obtained
- who signed the ICF
- if sufficient time has been given to consider participation into the study
- which investigator obtained ICF with the date of signature
- if a copy was provided to the patient
- start and end of participation in the study

## 15.3. Approval of the study protocol

### 15.3.1. General

The protocol has been reviewed and approved by the Ethics Committee of the Ghent University (Hospital), designated as the central Ethics Committee, after consultation with the local Ethics Committees, and the Federal Agency for Medicine and Health Products (FAMHP). This study cannot start before both approvals have been obtained.

### 15.3.2. Protocol amendments

Any significant change or addition to the protocol can only be made in a written protocol amendment that must be approved by the Central Ethics Committee (and the FAMHP if applicable).
Only amendments that are intended to eliminate an apparent immediate safety threat to patients may be implemented immediately.
Notwithstanding the need for approval of formal protocol amendments, the investigators are expected to take any immediate action, required for the safety of any subject included in this study, even if this action represents a deviation from the protocol. These actions should always be notified to the sponsor.

## 15.4. Confidentiality and Data Protection

All study data will be handled in accordance with the law on General Data Protection Regulation (GDPR) and institutional rules
[Belgian law dated on 30 July 2018 and 22 Aug. 2002].

The collection and processing of personal data from subjects enrolled in this study will be limited to those data that are necessary to fulfill the objectives of the study. These data must be collected and processed with adequate precautions to ensure confidentiality and compliance with applicable data privacy protection laws and regulations.

Appropriate technical and organizational measures to protect the personal data against unauthorized disclosures or access, accidental or unlawful destruction, or accidental loss or alteration must be put in place. Sponsor and site personnel whose responsibilities require access to personal data agree to keep the identity of subjects confidential.
The informed consent obtained from the subject includes explicit consent for the processing of personal data and for the investigator/institution to allow direct access to his or her original medical records (source data/documents) for study-related monitoring, audit, Ethics Committee review and regulatory inspection. This consent also addresses the transfer of the data to other entities, if applicable.

Privacy and confidentiality of data generated in the future on stored samples will be protected by the same standards applicable to all other clinical data.
Stored samples will be pseudonymized throughout the sample storage and analysis process and will not be labeled with personal identifiers.

## 15.5. Liability and Insurance

The sponsor has taken a no fault insurance for this study, in accordance with the relevant legislation (article 29, Belgian Law of May 7, 2004).
Sponsor: Ghent University Hospital
Insurance Details: Allianz Global Corporate & Specialty; Uitbreidingstraat 86, 2600 Berchem; Tel: +32 33 04 16 00
Polis number: BEL000862

## 15.6. End of Study Notification

If all subjects have completed the study, a notification of the end of the study should be submitted to the (Central) Ethics Committee and FAMHP. This notification should be made within 90 days of the end of the clinical study. In case of early termination (definition in CT-1, 4.2), this is reduced to 15 days.

# 16. Publication policy

This study will be registered at ClinicalStudies.gov, and results information from this study will be submitted to ClinicalStudies.gov. In addition, every attempt will be made to publish results in peer-reviewed journals.

# 17. Reference List

References


1. G. Dranoff, The Cloning of GM-CSF. *J Immunol* **198**, 2519-2521 (2017).
2. M. Guilliams *et al.*, Alveolar macrophages develop from fetal monocytes that differentiate into long-lived cells in the first week of life via GM-CSF. *J Exp Med* **210**, 1977-1992 (2013).
3. T. Naessens *et al.*, GM-CSF treatment prevents respiratory syncytial virus-induced pulmonary exacerbation responses in postallergic mice by stimulating alveolar macrophage maturation. *J Allergy Clin Immunol* **137**, 700-709 e709 (2016).
4. L. van de Laar *et al.*, Yolk Sac Macrophages, Fetal Liver, and Adult Monocytes Can Colonize an Empty Niche and Develop into Functional Tissue-Resident Macrophages. *Immunity* **44**, 755-768 (2016).
5. B. Mathias, B. E. Szpila, F. A. Moore, P. A. Efron, L. L. Moldawer, A Review of GM-CSF Therapy in Sepsis. *Medicine (Baltimore)* **94**, e2044 (2015).
6. J. J. Presneill, T. Harris, A. G. Stewart, J. F. Cade, J. W. Wilson, A randomized phase II trial of granulocyte-macrophage colony-stimulating factor therapy in severe sepsis with respiratory dysfunction. *Am J Respir Crit Care Med* **166**, 138-143 (2002).
7. R. Paine, 3rd *et al.*, A randomized trial of recombinant human granulocyte-macrophage colony stimulating factor for patients with acute lung injury. *Crit Care Med* **40**, 90-97 (2012).
8. S. Herold *et al.*, Inhaled granulocyte/macrophage colony-stimulating factor as treatment of pneumonia-associated acute respiratory distress syndrome. *Am J Respir Crit Care Med* **189**, 609-611 (2014).
9. R. Tazawa *et al.*, Inhaled granulocyte/macrophage-colony stimulating factor as therapy for pulmonary alveolar proteinosis. *Am J Respir Crit Care Med* **181**, 1345-1354 (2010).
10. R. Tazawa *et al.*, Inhaled GM-CSF for Pulmonary Alveolar Proteinosis. *N Engl J Med* **381**, 923-932 (2019).
11. Z. Xu *et al.*, Pathological findings of COVID-19 associated with acute respiratory distress syndrome. *Lancet Respir Med*, (2020).
12. E. S. Halstead *et al.*, GM-CSF overexpression after influenza a virus infection prevents mortality and moderates M1-like airway monocyte/macrophage polarization. *Respir Res* **19**, 3 (2018).
13. T. M. Umstead *et al.*, Lower respiratory tract delivery, airway clearance, and preclinical efficacy of inhaled GM-CSF in a postinfluenza pneumococcal pneumonia model. *Am J Physiol Lung Cell Mol Physiol* **318**, L571-L579 (2020).
14. B. Rosler, S. Herold, Lung epithelial GM-CSF improves host defense function and epithelial repair in influenza virus pneumonia-a new therapeutic strategy? *Mol Cell Pediatr* **3**, 29 (2016).
15. Y. Zhan, A. M. Lew, M. Chopin, The Pleiotropic Effects of the GM-CSF Rheostat on Myeloid Cell Differentiation and Function: More Than a Numbers Game. *Front Immunol* **10**, 2679 (2019).
16. C. Schneider *et al.*, Frontline Science: Coincidental null mutation of Csf2ralpha in a colony of PI3Kgamma-/- mice causes alveolar macrophage deficiency and fatal respiratory viral infection. *J Leukoc Biol* **101**, 367-376 (2017).
17. B. Unkel *et al.*, Alveolar epithelial cells orchestrate DC function in murine viral pneumonia. *J Clin Invest* **122**, 3652-3664 (2012).
18. F. F. Huang *et al.*, GM-CSF in the lung protects against lethal influenza infection. *Am J Respir Crit Care Med* **184**, 259-268 (2011).

19. G. M. Tse *et al.*, Pulmonary pathological features in coronavirus associated severe acute respiratory syndrome (SARS). *J. Clin. Pathol.* 57, 260-265 (2004).

# Appendices

## Appendix 1: USPI (US Package insert)
